# Orally Administrated Recombinant Vaccinia Virus Displaying ROP4 Induces Protection against *Toxoplasma gondii* Challenge Infection

**DOI:** 10.3390/vaccines10020152

**Published:** 2022-01-20

**Authors:** Keon-Woong Yoon, Ki-Back Chu, Hae-Ji Kang, Min-Ju Kim, Gi-Deok Eom, Fu-Shi Quan

**Affiliations:** 1 Department of Biomedical Science, Graduate School, Kyung Hee University, Seoul 02447, Korea; ky20200310318@khu.ac.kr (K.-W.Y.); heajik0514@khu.ac.kr (H.-J.K.); mj16441@khu.ac.kr (M.-J.K.); ekd3910@khu.ac.kr (G.-D.E.); 2 Department of Medical Zoology, School of Medicine, Kyung Hee University, Seoul 02447, Korea; kbchu@khu.ac.kr; 3 Medical Research Center for Bioreaction to Reactive Oxygen Species and Biomedical Science Institute, School of Medicine, Graduate School, Kyung Hee University, Seoul 02447, Korea

**Keywords:** recombinant vaccinia virus, ROP4, *T. gondii*, vaccine, ME49, mucosal immunity

## Abstract

Recombinant vaccinia viruses (rVVs) are attenuated viruses and are widely utilized as vectored vaccine platforms against numerous diseases. However, the protective efficacy of these rVV vaccines against *Toxoplasma gondii* and the resulting mucosal immunity has not been thoroughly assessed. Here, rVVs expressing the rhoptry protein 4 (ROP4) of *T. gondii* were generated. To evaluate the protection induced by the vaccines, mice were orally immunized with the ROP4-rVVs and subsequently challenge-infected with a lethal dose of *T. gondii* ME49 strain. Immunization with the rVVs induced higher levels of parasite-specific IgG and IgA antibody responses in sera compared to unimmunized control (NC). Upon challenge infection, significantly higher levels of IgG or IgA antibody responses in the brain, intestines, and vaginal samples were found in the immunized mice compared to NC. The ROP4-rVV vaccination elicited potent IgG and IgA secreting cell (ASC) responses, while substantially enhancing germinal center B cell, as well as CD4^+^ and CD8^+^ T cell responses from lymphoid organs. The production of pro-inflammatory cytokines IFN-γ and IL-6 in the brains was markedly diminished following immunization. The immunized mice also experienced reduced bodyweight loss and possessed fewer brain cysts than the control group. These results suggest that oral delivery of ROP4 displaying rVVs induced mucosal and systemic immunities that contributed to protection against lethal *T. gondii* challenge infection.

## 1. Introduction

Infection in humans can occur through the ingestion of undercooked meat and contaminated water, resulting in severe symptoms depending on the host’s immune status. In the case of immunocompromised individuals, pregnant women, and the elderly, it can cause congenital toxoplasmosis, resulting in hydrocephalus and microcephaly, and in severe cases miscarriage [1]. The current treatment options for *T. gondii* infection involve co-administration of pyrimethamine and sulfadiazine. Other drugs such as hydroxynaphthoquinone (atovaquone) and azithromycin could also be used, but there are major issues with these drugs. Pathogen drug resistance is a recurring theme frequently reported from various diseases and toxoplasmosis is no exception. This and the severe side effects, poor patient tolerability, and therapeutic ineffectiveness against bradyzoites of *T. gondii* are several of the obstacles that must be surmounted [1]. Vaccines are widely considered to be the most cost-effective healthcare intervention, and successful vaccines can benefit public health, which ultimately translates to economic growth [2]. For these reasons, developing a vaccine to protect against toxoplasmosis is an urgent need.

Vaccinia virus has been used extensively as a vaccine platform against numerous diseases such as smallpox. Currently, genetically modified vaccinia virus-based vaccines such as the modified vaccinia Ankara (MVA) are frequently used in heterologous prime-boost immunization strategies and have proven to be highly effective. To date, only a few studies have investigated the vaccine efficacies of recombinant vaccinia virus against toxoplasmosis. Roque-Resndiz et al. [3] reported that ROP2-expressing MVA vaccines were capable of inducing *T. gondii*-specific antibodies and that their immunization prolonged the survival of mice upon challenge infection with the RH strain. Heterologous immunization strategies involving MVA vaccines were also successful. In comparison to homologous immunization, heterologously immunizing GRA4-expressing DNA and MVA vaccines elicited greater brain cyst reduction and promoted longer survival of immunized mice after challenge infecting 20,000 PLK strains [4]. Similar findings were observed in another comparative study. Heterologously immunizing mice with SAG1-expressing adenovirus and MVA vaccines induced greater protection than homologous adenovirus-vectored vaccines [5].

The rhoptry proteins of *T. gondii* are parasitic components required for proliferation and host cell invasion [6,7]. Several studies have assessed the efficacy of ROP4 antigens using various vaccine platforms such as virus-like particles (VLPs) [8] or subunit vaccines [9]. Furthermore, we have recently investigated the efficacy of ROP4-expressing recombinant baculovirus (rBV) vaccines and the resulting immune responses in mice [10]. Nevertheless, there are inherent problems with the baculovirus-based vaccines. First and foremost, repeated vaccination with the baculovirus vaccines can result in the development of baculovirus-specific antibodies in hosts and subsequent neutralization of the baculovirus upon re-entry [11,12]. Another issue is the rapid degradation of baculoviral particles by the host complement system, thus limiting their application as vaccines [12,13]. To address these limitations of our previous works, we generated a ROP4-expressing recombinant vaccinia virus (rVV) vaccine as not a single study has investigated their efficacy in animal models. Orally administered vaccines are highly sought after due to their mucosal and systemic immunity-inducing effects [14]. Given that *T. gondii* is orally acquired in hosts, mucosal immunity is of importance since the intestinal mucosa acts as the initial site of infection for *T. gondii* and many other pathogens. Here, we investigated the resulting mucosal immunity induced by the ROP4-rVVs against *T. gondii* infection, which has yet to be reported. Our findings demonstrated that the ROP4-rVV vaccines elicited a robust cellular and humoral response that protected mice upon lethal *T. gondii* infection. 

## 2. Materials and Methods

### 2.1. Animals and Ethics

Six-week-old female BALB/c mice purchased from NARA Biotech (Seoul, Korea) were used for this study. The animals were housed in a specific-pathogen-free facility with easy access to food and water. All animal experiment protocols were approved and performed in accordance with the guidelines of Kyung Hee University IACUC (permit number: KHUASP (SE) 20-648). 

### 2.2. Parasite, Cells, and Antibodies

Parasites and cells used in this study were maintained as described earlier (Kang et al., 2020b). *T. gondii* ME49 strain was maintained by serial passaging in BALB/c mice. Mice were sacrificed and ME49 cysts were purified from the brain tissues of infected mice. CV-1 and Vero cells were cultured in T175 flasks with Dulbecco’s Modified Eagle’s Medium (ThermoFisher, Waltham, MA, USA) supplemented with 1% penicillin/streptomycin and 10% fetal bovine serum (FBS) at 37 °C. Horseradish peroxidase (HRP)-conjugated goat anti-mouse IgG and IgA secondary antibodies were purchased from Southern Biotech (Birmingham, AL, USA). 

### 2.3. Generation of Recombinant Vaccinia Virus (rVV)

The rhoptry protein 4 (ROP4) gene was amplified by polymerase chain reaction (PCR) and cloned into pRB21 vectors with StuI/HindIII restriction enzymes [15]. Prior to transfection with the recombinant pRB21 plasmid containing the ROP4 gene using Cellfectine II reagent (Invitrogen, Waltham, MA, USA), CV-1 cells were infected with the nonplaque-forming vRB12 mutant as previously described [16]. Infected cells were harvested with a scraper once morphological changes and other cytopathic effects were detected from approximately 70% of the seeded cells and centrifuged to pellet the cells. Supernatants were aspirated and cells were subjected to repeated freeze–thaw cycles that involved freezing at −80 °C in a deep freezer for 30 min immediately followed by thawing at 37 °C in a water bath a total of 3 times. After this procedure, cells were sonicated and centrifuged at 6000 rpm, 20 min, 4 °C. Supernatants were removed and pellets were resuspended in fresh DMEM media. To further purify the rVVs, resuspended rVV-containing cell pellets were carefully stacked on top of 36% sucrose gradient. After centrifugation at 20,000 rpm, 30 min, 4 °C, a distinct clear band was observed and this fraction was carefully collected. After resuspending the band fraction containing the rVV in 0.1 M PBS, protein concentration was determined using the QuantiPro BCA Assay Kit (Sigma-Aldrich, St Louis, MA, USA).

### 2.4. Quantification of rVVs by Plaque Assay

Vero cells were used to quantify rVV titers. In brief, confluent monolayers of Vero cells were cultured in 12-well plates in DMEM media supplemented with 1% penicillin/streptomycin and 10% FBS. Upon reaching 90–100% confluence, cells were infected with the serially diluted rVVs (10^2^, 10^3^, 10^4^ dilutions in serum-free media) and incubated at 37 °C, 5% CO_2_ for 1 h. Virus inoculum was removed after 1 h and overlay media (1% noble agar and 2% FBS in DMEM media) were gently added into each well. Plates were incubated at 37 °C for 4 days and enlarged viral plaques were counted to determine rVV titers. 

### 2.5. Immunization and Challenge Infection

A total of 18 mice were subdivided into 3 groups (*n* = 6 per group): unimmunized (Naïve), unimmunized mice that were challenge-infected (Naïve+Cha), and rVV-immunized mice. Mice were primed and boost-immunized with 5 × 10^3^ pfu/100 μL of ROP4-rVVs through the oral route by oral gavage at 4-week intervals. Four weeks after the boost immunization, mice were orally challenge-infected with a 50 LD_50_ dose (2000 cysts) of ME49 cysts. Mice were monitored daily to assess changes in bodyweight and survival rates. Mice that lost more than 20% of their initial bodyweight were humanely euthanized. At 16 days post-infection (dpi), all the mice were sacrificed for sample collection and immunological assays were performed.

### 2.6. Sample Collection and Preparation

Samples were collected as previously described [10]. Four weeks after each immunization, blood samples of mice were collected by retro-orbital plexus puncture. Sera were separated from the blood via centrifugation at 5000 RPM for 10 min. Sera were diluted (1:100 dilution in PBS) and used as primary antibodies for ELISA. After sacrificing all of the mice at 16 dpi, brain tissues and other mucosal samples were acquired. Briefly, 100 μL of 0.1 M PBS was added per 0.1 g of fecal sample. Vaginal samples were collected by washing the vaginal canals with 200 μL of 0.1 M PBS. Intestinal samples were harvested by longitudinally sectioning the intestines and immersing the tissues in 500 μL of 0.1 M PBS. Mucosal samples were incubated at 37 °C for 1 h then centrifuged at 500 rpm for 10 min. Supernatants were collected and samples were stored at −20 °C until use. The brain tissues were used to quantify cyst burden in mice. Spleens and mesenteric lymph nodes were carefully harvested for immunological assays. All the samples were processed on an individual basis.

### 2.7. Enzyme-Linked Immunosorbent Assay (ELISA) and Antibody-Secreting Cell (ASC) Responses

*T. gondii*-specific serum antibody responses were determined as previously described [8,10]. To evaluate ASC induction, splenic and mesenteric lymph nodes (MLNs) were collected from mice. Single cell suspensions of splenocytes and MLN cells were prepared as previously described [8]. After RBC lysis, splenocytes and MLN cells were seeded into 96-well immunoplates coated with *T. gondii* ME49 whole antigen (4 μg/mL). Plates were incubated for 5 days at 37 °C. Supernatants were removed and HRP-conjugated anti-mouse IgG or IgA antibodies were added into each well. Plates were incubated for 1 h at 37 °C, and 100 μL of OPD substrate dissolved in citrate substrate buffer were inoculated into each well. Reactions were stopped using dilute H_2_SO_4_ and OD_490_ values were measured.

### 2.8. Flow Cytometry and Immune Cell Population Assessment

Flow cytometry was performed on splenocytes and MLN cells as previously described [17]. Splenocytes (1 × 10^6^ cells/mouse) and MLN cells (1 × 10^5^ cells/mouse) were stimulated with *T. gondii* ME49 antigen (2 μg/mL) at 37 °C with 5% CO_2_ for 2 h. Stimulated cells were stained with fluorophore-conjugated antibodies purchased from BD Biosciences (Franklin Lakes, NJ, USA) and Invitrogen (Waltham, MA, USA). CD4^+^ T cells, CD8^+^ T cells, and germinal center B (GC B) cell populations were measured using the following antibodies: CD3 (PE- Cy7), CD4 (FITC), CD8 (PE), GL7 (PE), and B220 (FITC). All staining procedures were performed according to the manufacturer’s protocol. Stained cells were acquired by Accuri C6 flow cytometer and analyzed with the C6 Accuri software (BD Biosciences, Franklin Lakes, NJ, USA). For GC B cells, B220^+^ lung cells and splenocyte populations were gated, and from this population those positive for GL7 were gated. For CD4^+^ and CD8^+^ T cells, CD3-positive single cell populations were gated and from this population CD4^+^ and CD8^+^ cells were determined.

### 2.9. Inflammatory Cytokine Production and Cyst Burden in the Brain

Brain tissues of individual mice were subdivided into left and right hemispheres and processed separately for inflammatory cytokine assessment and cyst count, respectively. The left hemispheres were homogenized in 500 μL of PBS and then centrifuged at 10,000 rpm for 10 min. Supernatants were carefully collected and stored at −80 °C until use. Pro-inflammatory cytokines in these supernatants were measured using IFN-γ and IL-6 BD OptEIA ELISA kits (BD Biosciences, Franklin Lakes, NJ, USA). All experiments were performed according to the manufacturer’s instructions and standard curves were generated to calculate the cytokine concentrations. The right hemispheres of the brains were used to quantify cyst burdens in challenge-infected mice following the method previously described [18]. Tissues were homogenized in 500 μL of PBS and centrifuged at 12,100 rpm for 20 min through Percoll density gradient media (BD Biosciences, Franklin Lakes, NJ, USA). The *T. gondii* cyst layer was carefully collected and repeatedly washed with PBS. Cysts from each sample were counted 3 times from 3 different fields of view under a microscope (Leica DMi8, Leica, Wetzlar, Germany).

### 2.10. Statistical Analysis

All statistical analyses were performed using Graphpad Prism version 6 (San Diego, CA, USA). A one-way ANOVA with Tukey’s post hoc test or 2-way ANOVA with Bonferroni’s post hoc test was performed to assess the statistical significance between the means. Data are presented as mean ± SD and statistical significance is denoted with asterisks. *p* values of * *p* < 0.05, ** *p* < 0.01, *** *p* < 0.001, and **** *p* < 0.0001 were considered statistically significant.

## 3. Results

### 3.1. Vaccinia Virus Characterization

**Figure 1:**
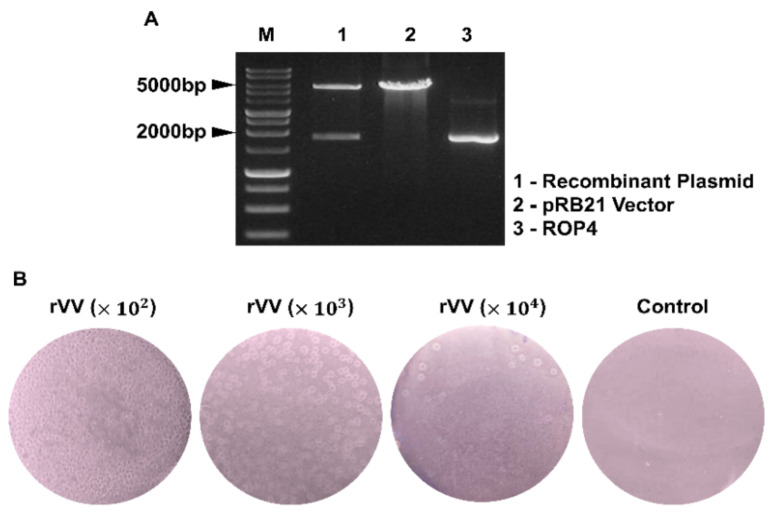
Recombinant vaccinia virus characterization. Recombinant plasmid constructs containing the *T. gondii* ROP4 gene were cleaved with the restriction enzymes. The size of the ROP4 gene and the pRB21 vectors were 1761 bp and 5537 bp, respectively (M: Marker, Lane 1: pRB21 + ROP4, Lane 2: pRB21, Lane 3: ROP4) (**A**). Virus titers of the ROP4-rVV were determined by plaque assay in Vero cells. Plaques were visualized under a microscope at 4 dpi. Representative images from 3 independent experiments performed in triplicates were acquired at 100× magnification (**B**).

The *T. gondii* ROP4 gene was PCR-amplified and subsequently cloned into the pRB21 vector. To confirm the successful integration of the ROP4 gene into the pRB21 vector, restriction enzyme digestion with StuI and HindIII was performed (Figure 1A). Virus titers of the rVVs were determined by plaque assay. Distinct white plaques were detected from the Vero cells infected with the serially diluted rVVs (10^2^, 10^3^, 10^4^ dilutions). Plaque counts were inversely proportional to the dilution fold, with the highest number of plaques being observed from 10^2^ dilutions. No plaques were observed from the vehicle control group (Figure 1B).

### 3.2. IgG and IgA Antibody Response in Sera and Mucosal Samples

**Figure 2:**
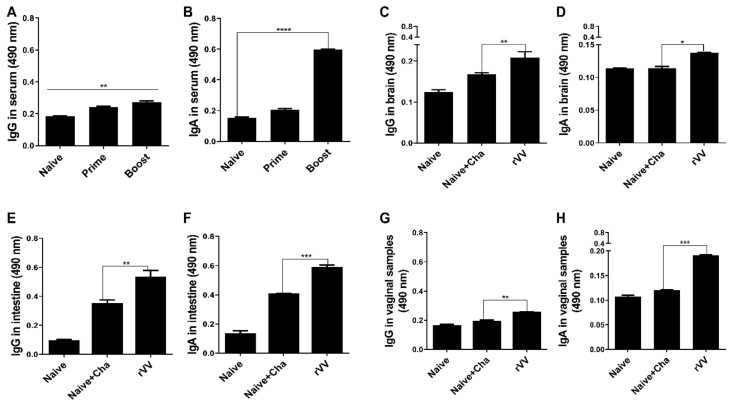
Evaluating serum and mucosal antibody inductions. BALB/C mice (*n* = 6 per group) were immunized with the ROP4-rVVs by the oral route. Sera were collected 4 weeks after immunization, and *T. gondii*-specific sera IgG and IgA responses to *T. gondii* ME49 antigen were determined by ELISA (**A**,**B**). *T. gondii*-specific mucosal IgG and IgA responses in the brain (**C**,**D**), intestines (**E**,**F**), and vaginal secretions (**G**,**H**) were collected at 16 dpi to evaluate mucosal antibody responses. Data are presented as mean ± SD from 3 independent experiments performed in triplicates and asterisks indicate statistical differences between groups. (* *p* < 0.05, ** *p* < 0.01, *** *p* < 0.001, **** *p* < 0.0001).

The *T. gondii*-specific antibody responses were measured using the sera acquired from mice after prime and boost immunizations. Compared to unimmunized control, immunization with the ROP4-rVV elicited antibody responses. Specifically, increasing the number of immunizations enabled greater production of both IgG and IgA in the sera. In stark contrast to the IgG responses, a drastic increase in IgA response was observed after boost immunization (Figure 2A,B). At 16 dpi, mucosal tissue samples were harvested and antibody responses were assessed. Similar to the serum ELISA data acquired after the two immunizations, antibody inductions in the mucosal tissues were also observed after the challenge infection. Compared to the Naïve+Cha group, significant differences in brain IgG and IgA levels were detected (Figure 2C,D). An identical trend was also observed from the intestinal samples, which was characterized by robust IgG and IgA responses from the immunized mice (Figure 2E,F). Marginal increases in antibody responses were also detected from the vaginal secretions of mice (Figure 2G,H). Expectedly, mucosal antibody responses were induced by oral immunization with ROP4-rVVs in mice.

### 3.3. Antibody Secreting Cell Response

**Figure 3:**
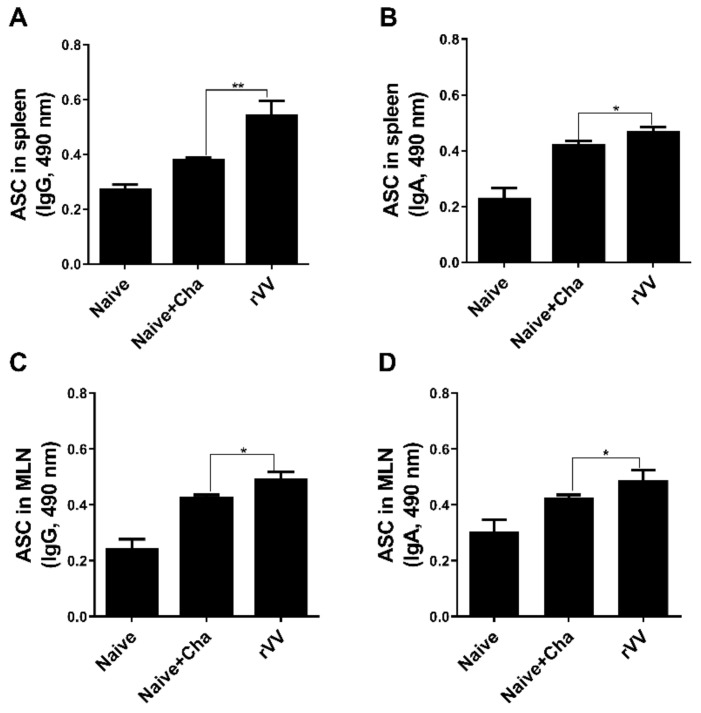
Antibody-secreting cell responses in secondary lymphoid organs. At 16 dpi, mice (*n* = 6 per group) were sacrificed and the levels of IgG and IgA antibody-secreting cells were evaluated in spleen and MLN cells. Single-cell populations of splenocytes and MLN cells were incubated with *T. gondii* ME49 lysate antigen. Splenic IgG and IgA (**A**,**B**, respectively) and MLN IgG and IgA (**C**,**D**, respectively) were evaluated by ELISA. Data are presented as mean ± SD from 3 independent experiments performed in triplicates and asterisks indicate statistical differences between groups. (* *p* < 0.05, ** *p* < 0.01).

To determine antibody-secreting cell responses, splenocytes and MLN cells were collected from mice and cultured for 5 days with *T. gondii* antigens. Compared to both unimmunized control and Naïve+Cha mice, splenic IgG ASC responses were significantly higher in the immunized mice (Figure 3A). While this was also the case with splenic IgA ASC, the differences between the means of Naïve+Cha and ROP4-rVV were not as drastic as those of IgG (Figure 3B). ASC responses in the MLN were similar to those demonstrated by splenocytes. Immunization with the rVVs elicited significantly greater quantities of IgG and IgA ASC responses than Naïve+Cha (Figure 3C,D). 

### 3.4. Germinal Center B Cell Response in Spleen and MLN

**Figure 4:**
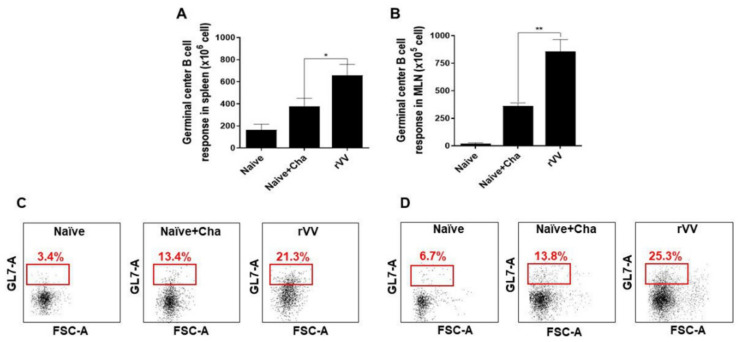
ROP4-rVV immunization induces potent GC B cell responses. Spleens and MLNs were harvested from mice (*n* = 6 per group) at 16 dpi and GC B cell proliferation was assessed by flow cytometry. GC B cell populations from antigen-stimulated spleen (**A**) and MLN (**B**) were evaluated. Representative scatterplots for both spleen (**C**) and MLN (**D**) were provided, with boxes indicating the gated GC B cell population. Data are presented as mean ± SD from 3 independent experiments performed in triplicates and asterisks denote statistical differences between groups (* *p* < 0.05, ** *p* < 0.01).

Vaccine-induced immune responses were further assessed using flow cytometry. At 16 dpi, compared to Naïve+Cha control group, splenic GC B cell populations were nearly doubled in the ROP4-rVV immunization group (Figure 4A). An identical trend was also observed with MLN as well (Figure 4B). A scatterplot depicting GC B cell populations from both splenocytes and MLN (Figure 4C,D) revealed that ROP4-rVV immunization contributed to high levels of GC B cell induction in both lymphoid organs.

### 3.5. Activation of CD4^+^ and CD8^+^ T Cells in MLN

**Figure 5:**
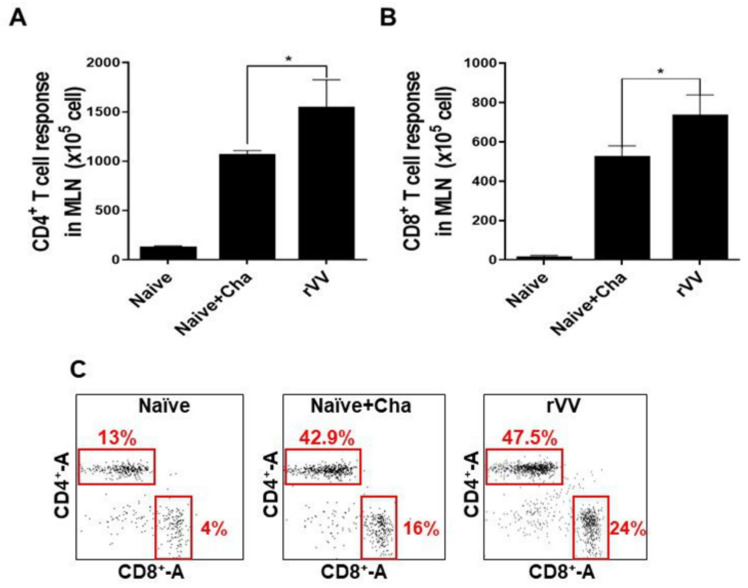
ROP4-rVV enhances T cell inductions in the MLNs of immunized mice. MLN cells of mice were collected. Single-cell populations of MLN cells were stimulated with *T. gondii* ME49 antigens. CD4^+^ (**A**) and CD8^+^ (**B**) T cells were quantified using flow cytometry. Representative scatterplots illustrating lymphocyte gating of the CD4^+^ and CD8^+^ T cells from 3 independent experiments were provided (**C**). Boxes indicate the percentages of both CD4^+^ and CD8^+^ T cells. Data are presented as mean ± SD from 3 independent experiments and asterisks denote statistical differences between groups (* *p* < 0.05).

CD4^+^ and CD8^+^ T cell activity was measured in mesenteric lymph nodes. Drastically increased CD4^+^ T cell populations were observed from Naïve+Cha and ROP4-rVV immunization groups, with CD4^+^ T cell induction being significantly greater in the latter (Figure 5A). Profound CD8^+^ T cell proliferation was also detected from the MLNs of Naïve+Cha and ROP4-rVV groups (Figure 5B). Gated T cell populations for each group were illustrated using a scatterplot (Figure 5C). Representative plots reveal that challenge infection with the 50 LD_50_ *T. gondii* ME49 enhanced CD4^+^ and CD8^+^ T cell populations by roughly 30% and 12%, respectively. These values were further enhanced if the mice were immunized with the ROP4-rVVs.

### 3.6. Pro-Inflammatory Cytokine Response

**Figure 6:**
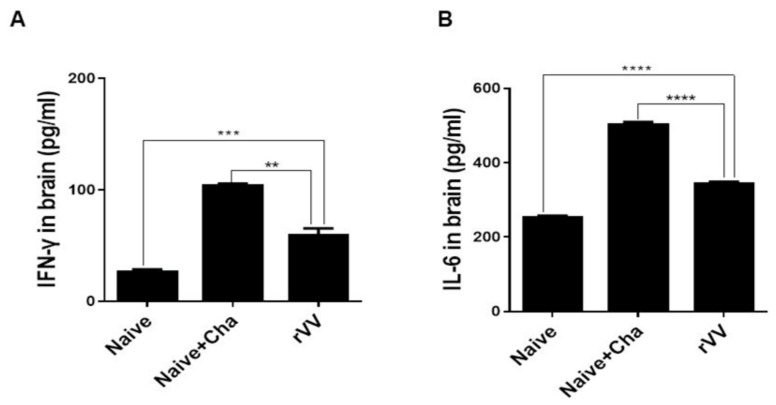
Immunization with ROP4-rVV vaccine inhibits inflammatory cytokine production. Brain tissues were isolated from mice and tissue homogenates were used to detect cytokines IFN-γ (**A**) and IL-6 (**B**) by ELISA. Data are presented as mean ± SD from 3 independent experiments performed in triplicates and asterisks indicate statistical differences between groups (** *p* < 0.01, *** *p* < 0.001, **** *p* < 0.0001).

To determine the amount of pro-inflammatory cytokines, IFN-γ and IL-6 levels in the brain homogenates were measured. Compared to the Naïve control, large quantities of inflammatory cytokines were produced in the brain tissues of Naïve+Cha mice. Vaccination with the ROP4-rVV ameliorated the inflammatory response by significantly reducing the production of IFN-γ (Figure 6A) and IL-6 (Figure 6B) in the brains of mice. 

### 3.7. Protective Efficacy of the ROP4-rVV Vaccine

**Figure 7:**
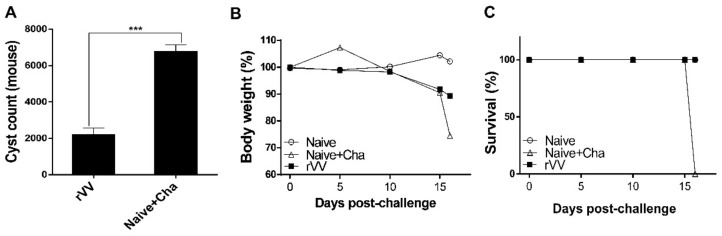
**The** ROP4-rVV vaccine protects mice against *T. gondii* ME49. The protective efficacy of the vaccine was evaluated by assessing cyst counts, bodyweight reduction, and survival. Cysts were counted from the right hemispheres of mice (*n* = 6 per group) under the microscope (**A**). Mice were monitored daily to check changes in bodyweight (**B**) and survival (**C**). Data are presented as mean ± SD from 3 independent experiments and asterisks indicate statistical differences between groups (*** *p* < 0.001).

To confirm the protective efficacy of ROP4-rVV against *T. gondii* ME49 infection, brain cysts were counted under the microscope. More than 6000 cysts were counted in Naïve+Cha, whereas the immunized mice had approximately a threefold decrease in the number of parasitic cysts (Figure 7A). All Naïve+Cha mice lost more than 20% of their initial bodyweight and were humanely euthanized at 16 dpi with a lethal dose of ME49. Contrary to these results, ROP4-rVV immunization ensured that mice maintained bodyweights close to the normal range (Figure 7B). Although all of the Naïve+Cha mice perished, none of the immunized mice showed signs of illness, and 100% survival was observed (Figure 7C). These results indicate that administering the ROP4-rVV vaccine via the oral route is effective against *T. gondii* ME49 infection.

## 4. Discussion

Oral vaccines are highly desired for several reasons. They are easy to administer with excellent patient compliance since pain infliction is virtually non-existent. Since these vaccines do not require the presence of highly trained medical personnel for proper administration, it is an efficient mass vaccination strategy [19]. Nevertheless, the major obstacle with mucosal immunization via the oral route is the need to traverse through the highly acidic environment of the stomach. Consequently, antigenic epitopes are prone to degradation and this substantially lessens the efficacy of the vaccines [19]. While the ROP4-rVV vaccine used in the present study was subjected to such detrimental effects, they were immunogenic and conferred protection against a lethal dose of *T. gondii* ME49 challenge infection in mice.

Most of the immunological findings reported here are similar to the protective efficacy induced by ROP4-rBV vaccines as described in our previous study, although several differences in immunological parameters were noted [10]. Consistent with our previous study, a remarkable increase in GC B cells was observed from both the spleens and MLNs of mice. However, discrepancies were observed in the T cell populations. In the oral ROP4-rBV immunized mice, differences were not significant compared to the control groups, while such differences were observed in the ROP4-rVV immunized mice. Differences were also detected in terms of brain inflammatory cytokine production. While significant IL-6 reductions were not observed in our earlier works with the ROP4-rBVs, drastic IL-6 and IFN-γ reductions were observed in the current study. Although cyst counts were comparable for the two vectored vaccines in our works, bodyweight reductions were less severe when mice were immunized with the ROP4-rVVs. Given these results, the protection induced by the ROP4-rVV vaccine appears to be superior to that of the rBV vaccine expressing an identical antigen, thereby suggesting that rVV vectors serve as potent inducers of mucosal immunity in mice. In support of this notion, one study comparing the efficacies of MVA-based vaccines against rBV vaccines has reported similar findings. MVA vaccines expressing the VP2 antigen of African horse sickness virus conferred better protection in mice than rBV vaccines expressing the same antigen at an identical dosage and number of immunizations [20].

Antibodies are functionally required for mounting optimal protection against pathogens. Our findings revealed that immunization with the ROP4-rVV elicited significantly enhanced antibody responses in sera and also in the mucosal tissues. Mucosal antibodies, particularly the IgA subclass, are of importance. These antibodies deter the attachment of microorganisms to the mucosal surface [21]. Since the intestinal epithelial cells are the site of infection for *T. gondii*, mounting a strong first line of defense in these regions is important. The presence of these vaccine-induced mucosal antibodies, especially IgA, is crucial as they will act to limit parasitic entry into the host cells. Evidently, *T. gondii*-specific secretory IgA antibodies collected from patients were reported to have successfully inhibited the *T. gondii* infection of enterocytes [22]. As exemplified using these clinical samples, the ROP4-specific mucosal IgA elicited by the ROP4-rVV vaccine may have protected mice against challenge infection by limiting the parasitic entry into intestinal epithelial cells. Similarly, pro-inflammatory cytokines such as IFN-γ are essential for resistance against *T. gondii*, as their production by T cells is required to inhibit tachyzoite proliferation in the brain [23,24]. Nevertheless, this comes at the expense of neuronal damage. For instance, in pneumococcal meningitis mice models, reduced neuronal damages were observed from IFN-γ ablated mice whereas brain pathologies were much more severe in the wild-type mice [25]. Therefore, a balance in inflammatory cytokines that enable parasitic inhibition while inflicting minimal neuronal damage is necessary.

There were several discrepancies between our findings and those of another study. Surprisingly, immunizing C57BL/6 mice with the recombinant vaccinia virus vaccine (LC16m0 strain) expressing the GRA4 antigen of *T. gondii* failed to elicit antigen-specific antibody responses even after 3 immunizations at 3-week intervals [4]. Contrary to these results, profound antibody responses were observed in the sera and mucosal tissues of the ROP4-rVV immunized mice. Similar to our results, mice immunized with the ROP2-expressing recombinant MVA vaccine also developed high titers of antigen-specific antibody responses [3]. Identifying the exact factor accountable for such differences is unknown and difficult to discern, but it is possible that antigen candidates and the strain of the vaccinia virus may be involved. LC16m8 strain, which is derived from the LC16m0 strain, is categorized under 3rd generation vaccinia virus vaccines along with the MVA strain. However, their method of production and the extent of their attenuation greatly differs. Consequently, different strains of these 3rd generation vaccines were reported to possess differing virus properties, which include replication competency in mammalian cells, pro-inflammatory gene induction, and others.

Vaccinia virus dissemination into the ovaries of infected mice following intravenous and intraperitoneal inoculation has been reported, but such a phenomenon was not observed when the viruses were injected via a subcutaneous route [26]. Intranasally inoculating vaccinia virus resulted in uncontrolled virus replication in the murine lungs, which ultimately led to their death [27]. However, such issues were not observed from our study or by others [28] when vaccinia viruses were inoculated via oral routes, thus implying that the oral route of administration could be an effective way for developing vaccinia virus-based vaccine candidates. As previously mentioned, antigen degradation and efficacy loss is one detriment associated with oral vaccines. To overcome this issue and improve the efficacy of orally administered mucosal vaccines, alternative strategies could be employed. One such method would be immunization through the sublingual/buccal tissues outlining the oral cavity. Evidently, sublingual/buccal immunization with the MVA-based human immunodeficiency virus 1 (HIV-1) elicited potent mucosal immune responses and protected rhesus macaques upon challenge infection [29]. Another strategy would be to employ heterologous immunization as described through earlier MVA-based *T. gondii* vaccine studies [4,5]. 

One major limitation of this study is the lack of data confirming long-term protection. In our study, all mice were sacrificed at an identical time point to enable accurate comparison of immunological parameters between groups. Thus, long-term protection could not be evaluated. Therefore, interpreting the findings illustrated in Figure 7B would be speculative. For example, based on the downward trend of bodyweight, mice could either recover and survive for months or succumb to death via delayed disease progression. As such, further studies assessing this must be conducted. 

## 5. Conclusions

In summary, we demonstrated that orally immunizing mice with the ROP4-rVV vaccine induces robust systemic and mucosal immunity that fully protects mice from lethal challenge infection with *T. gondii* ME49. The live attenuated vaccine design strategy presented in this study may be suitable for the development of a safe and efficacious *T. gondii* vaccine.

## Data Availability

Data supporting the findings of this study are contained within the article.
